# A debate about ultrasound and anatomic aspects of the cervix in spontaneous preterm birth

**Published:** 2016

**Authors:** RE Bohîlțea, O Munteanu, N Turcan, A Baros, O Bodean, D Voicu, MM Cîrstoiu

**Affiliations:** * ”Carol Davila” University of Medicine and Pharmacy, Bucharest, Romania; ** University Emergency Hospital Bucharest, Romania

**Keywords:** cervical length, cervical screening, progesterone, cerclage, preterm birth

## Abstract

Preterm birth is the legal first global cause of neonatal death. The cervix has two roles: it has to stay closed to allow the fetus to undergo a normal development during gestation, and at term, the cervix has to dilate under the pressure of uterine contractions to allow the delivery. The purpose of this article is to establish if the ultrasound measured length of the cervix and its appearance are predictive for the spontaneous preterm birth. Cervical insufficiency can be described by painless cervical dilatation leading to pregnancy losses/ births, with no other risk factors present. During gestation, the physiological softening of the cervix is determined by the extracellular matrix components, particular decorin, and thrombospondin 2. The direction of the collagen fibers remains the same – circumferential direction, but the collagen solubility increases. Therefore, during pregnancy, the cervical tissue is more hydrated and has higher collagen extractability than non-pregnant tissue. Women with cervical incompetence have increased levels of smooth muscle cells than normal pregnant women, the number of elastic fibers is low, and also the concentration of hydroxyproline is decreased. Transvaginal ultrasound is the suitable gold standard exam that can offer essential information about the cervical length and state of the internal os in early asymptomatic stage of cervical insufficiency for predicting and preventing preterm birth. In our experience, a transvaginal ultrasound screening for the measurement of the cervix is required. We consider that the proper gestational age for the prediction of a preterm birth is at 18-22 weeks of gestation for the general population and earlier for patients with a history of preterm birth. Just from an observational point of view, we concluded with the fact that the cerclage of the cervix is unnecessary if the cervical length is above 2 cm and if the internal cervical os is closed. In the absence of funneling, the probability of cervical incompetence is low and the best prophylactic option is progesterone administration.

## Introduction

Preterm birth is the legal first global cause of neonatal death [**1**]. The purpose of this article was to establish, by reviewing the literature, if the ultrasound measured length of the cervix and the funneling are predictive for the spontaneous preterm birth and reliable for the selection of the proper prophylactic attitude. This is a highly discussed subject and numerous studies of various designs have evaluated the cervical length measurement in order to predict preterm birth in a variety of populations. A cervical length screening for all the pregnant women was suggested numerous times, in order to predict and prevent preterm birth, but neither the 2013 Cochrane review, nor the others had found sufficient evidences to recommend it by routine [**2**].

Multiple risk factors are related to spontaneous preterm birth, some of which can be identified and prevented, but many preterm births occur among women with no risk factors.

Cervical insufficiency can be described by a painless cervical dilatation leading to pregnancy losses/births, with no other risk factors present [**3**]. Cervical shortening is a known risk factor for preterm birth in both low- and high-risk populations. The most frequently encountered risk factors that can lead to cervical shortening and by default, pregnancy loss or preterm birth, are: decidua inflammation/infection, hemorrhage and uterine over distension, factors that can initiate biochemical changes in the cervix [**3**]. Cervical trauma, inadequate cervical dilatation prior to a gynecologic procedure and a therapeutical resection for various degrees of intraepithelial neoplasia are responsible for acquired cervical abnormalities, which, along with genetic disorders of collagen tissue, congenital uterine anomalies, and in utero exposure to diethylstilbestrol, increase the risk for cervical insufficiency. Past obstetrical history of the women with cervical insufficiency is important and in most cases suggestive, and antecedents of mid-trimester pregnancies loss or preterm deliveries, or progressively later preterm deliveries in successive pregnancies could characterize it. However,there are various reported cases of women with no risk factors and no suggestive obstetrical history, in whom the ultrasound exam revealed a short length of the cervix.

In normal cases, cervical length presents no modification between 14 and 28 weeks of gestation [**4**], its length being quantified as it follows:

●15 mm – 2nd centile

●20 mm – 5th centile

●25 mm – 10th centile

●35 mm – 50th centile

●45 mm – 90th centile

After 28 to 32 weeks of gestation, gradual declines in cervical length are normal. Cervical length is not affected by parity, race/ ethnicity or maternal height [**5**].

A short cervix can be defined by transvaginal ultrasound, the diagnosis being confirmed if the measured length of the cervix is ≤20 mm in women with no prior preterm delivery and <25 mm in women with a prior preterm delivery [**6**,**7**]. The prior preterm delivery is included in the definition of short cervix, because the evidence supporting various interventions is based upon both cervical length and prior pregnancy history. A 2011 meta-analysis of randomized trials found that vaginal progesterone supplementation prolonged pregnancy in women with ultrasound detected short cervix, and may be as effective as cerclage [**8**]. Women with a prior preterm birth and the cervical length below 25 mm have an indication for treatment with hydroxyprogesteronecaproate or vaginal progesterone and performance of cerclage.

## Sonographic assessment of the cervix 

Transabdominal ultrasound has multiple limitations regarding the cervical length assessment. The factors that embarrass an appropriate measurement of the cervical length are the following: the increased distance from the probe to the cervix, the bladder needs to be sufficiently filled for a reliable image to be produced, leading to elongation of the cervix and camouflaging of any funneling at the internal os, and the likelihood that fetal parts will obscure the cervix is higher, especially after 20 weeks of gestation. Transabdominal ultrasound should be used for the diagnosis of short cervix only after the other method has been taken into account [**9**].

Normally, an ultrasound exam can reveal changes of cervical aspect weeks before an eventual labor. Transvaginal ultrasound (TVU) is the gold standard suitable exam that can offer essential information about the cervical length in the early asymptomatic stage of cervical insufficiency for predicting and preventing preterm birth. Transvaginal sonography is a safe and acceptable method of studying the cervix, as it is well accepted by >99% of the women, and pain is reportedly felt in <2% of the cases [**10**]. Cervical changes visible on TVU include the initial opening of the internal cervical os, progressive cervical widening and shortening of the endocervical canal from internal to the external os, and finally dilation of the external os. Additional findings associated with preterm birth may be observed during the transvaginal ultrasound examination: separation of the membranes from the decidua and debris/sludge, representing blood clot, meconium, vernix, or cellular material related to the infection/inflammation. The presence of the findings described suggests a subclinical infection or inflammation that increases the risk of preterm birth [**11**].The opening of the internal cervical os allows the protrusion of the amniotic membranes into the cervical canal, process called funneling. As the cervix effaces, the relationship between the lower uterine segment and the axis of the cervical canal also changes and is described according to the shape of the letters “T”, “Y”, “V”, and “U”. “T” represents the normal relationship of the area where the endocervical canal meets the uterine cavity, whereas “U” represents almost complete effacement of the cervix and signifies the highest risk for preterm birth [**12**].

An underestimation of the cervical length is possible due to the excessive pressure placed on the cervix during the examination; the measurement of the cervix done too quickly does not allow enough time to view dynamic changes, contractions present during the examination being capable of leading to an erroneous impression of a long cervix. Also, as the underdevelopment of the lower uterine segment, placenta praevia can create difficulties in differentiating the limit of the endocervical mucosa, creating a false increased cervical length appearance [**13**].

## Cervical length screening

As already mentioned, a 2013 Cochrane review did not find sufficient evidence to recommend routine cervical length screening for all pregnant women. A clear protocol is required for the management of women based on their transvaginal measured cervical length. This protocol has to include characteristics that could affect the performance of the test such as the proportion of singleton versus multiple gestations, symptomatic versus asymptomatic women, intact membranes versus ruptured membranes, prior preterm birth versus no prior preterm birth, and prior cervical surgery versus no prior cervical surgery [**14**].

The sensitivity of short cervix for subsequent preterm birth in women with a singleton gestation and no prior preterm birth is approximately 35-40%, and the positive predictive value is approximately 20-30%. This means that the majority of singleton women with a short cervix will deliver in ≥35 weeks. Sensitivity increases to 70% in women with a prior preterm birth, and even higher in women with early and/or repeated preterm birth. The number of fetuses has a great impact on the cervical length. Women carrying twins are approximately twice as likely to have a short cervix at 24 to 28 weeks of gestation as women carrying singletons: 40 to 50 percent of twin pregnancies with cervical length ≤ 25 mm at 24 to 28 weeks deliver <35 weeks of gestation [**7**].

Three most prestigious guidelines from the national organizations do not approve cervical length screening to be universally mandated. The Society for Maternal-Fetal Medicine (2012) sustains as reasonable the implementation of such a screening strategy that can be considered by individual practitioners, following strict guidelines [**16**]. The American College of Obstetricians and Gynecologists has recommended that the cervix has to be examined when clinically appropriate and technically possible; a 2012 practice bulletin neither mandates universal routine cervical length screening in women without a prior preterm birth, nor recommends against such a screening [**17**,**18**]. The Society of Obstetricians and Gynecologists of Canada (SOGC) concluded in 2011 that a routine transvaginal cervical length assessment is not indicated in women at low risk [**19**]. The universal screening by transvaginal measurement of the cervix is not routinely recommended by the International Society of Ultrasound in Obstetrics and Gynecology (ISUOG) [**20**] and also not by the Romanian Society of Ultrasound in Obstetrics and Gynecology (SRUOG). The algorithm of Berghella sustains the transvaginal measurement of the cervix between 14/16 - 24 weeks of pregnancy as a screening method in patients without a medical history of preterm birth and also as a follow up at 2 weeks and weekly in patients with a history of preterm birth and the cervix length of ≥ 30mm and < 30mm respectively. The performance of cervical cerclage at 12- 14 weeks of pregnancy is indicated in patients with a medical history of preterm birth and cervix length < 25mm, and the progesterone administration until the 36thweek includes all the patients with a history of abortion in the second trimester or of spontaneous preterm birth, as well as the ones without a medical history but with the cervical length of ≤ 20mm [**21**]. 

## Anatomic considerations about cervical structure

There are many causes for preterm births, but the mechanical failure of the cervix is the common cause for multiple etiologies and maintaining a healthy gestation is mandatory to have a mechanical integrity of the cervical tissue [**22**].

An important risk factor for preterm birth is cervical incompetence/insufficiency - painless dilation and the shortening of the cervix in the second trimester of pregnancy [**23**],being responsible for preterm births before 28 weeks of gestation [**24**].

The cervix has two roles: it has to stay closed to allow the fetus to undergo a normal development during gestation, and at term, the cervix has to dilate under the pressure of uterine contractions to allow delivery [**25**].

Uterine cervix differs histologically from the rest of the uterus and these two autonomous parts of the same organ allow a synergistically efficient parturition [**26**]. The uterine cervix is composed of a musculofibrous central ax – fibrous connective tissue (85%, collagen, glycosaminoglycans, and extracellular matrix) and the smooth muscle (15% of which does not appear to contribute to the cervical strength) [**27**]. The ultrastructure of the collagen fiber is a major component of the mechanical function of the cervix during gestation; therefore, the strength of the cervical tissue is related to the content of collagen. The collagen network contains collagen type III and I; hydroxyproline (HOP) is a major component of the protein collagen that plays a key role in collagen stability [**8**]. Anum et al. affirm that the improper function and remodeling of the extracellular matrix may contribute to preterm birth and prematurity due to cervical incompetence [**28**]. However, Warren et al. demonstrated that TGF-β Arg-25-Pro polymorphisms are associated with this condition [**29**].

During gestation, the physiological softening of the cervix is determined by the extracellular matrix components, particular decorin, and thrombospondin 2. The direction of the collagen fibers remains the same – circumferential direction, but the collagen solubility increases [**30**]. Therefore, during pregnancy, the cervical tissue is more hydrated and has higher collagen extractability than non-pregnant tissue [**31**].

Myers et al. observed that the added resistance to the circumferential strain is a protective feature of the cervix dilatation and over–stretching of the interspersed cells that may control cervical remodeling [**32**].

Rath et al. state that there is an increased level of hyaluronic acid during pregnancy, more frequently near delivery and it has been related to an increased collagen solubility and disorganization of the collagen network[**33**].

Women with cervical incompetence have increased levels of smooth muscle cells than normal pregnant women, the number of elastic fibers is low, and also the concentration of hydroxyproline is decreased [**34**]. Hydroxyproline and proline permit the sharp twisting of the collagen helix and HOP is found in few proteins, other than collagen, therefore, the quantification of hydroxyproline is a recognized method for theevaluation of cervical collagen [**35**].

Gedikbasi et al. affirm that a decreased expression of collagen may be associated with cervix remodeling during the first trimester of pregnancy and cervical collagen concentration is lower in women with cervical incompetence [**36**].

In another experimental study, Myers et al. demonstrated that the mechanical properties of cervical tissue may be severely modified by relatively small modifications in its biochemistry: higher collagen extractability and hydration levels are related to a more compliant tissue and also the total collagen content remains unchanged, but, there are significant changes in collagen crosslinking and in the quantity of glycosaminoglycans and hyaluronic acid [**37**].

Manabe et al. affirm that the uterus has multiple pacemakers in different locations, and the uterine distention works as a trigger for the elevation of excitability and for coordinated and orthodromic activities [**38**]. The study group coordinated by Karsdon demonstrated that it is safe and feasible to treat human preterm uterine contractions with a weak electrical current [**39**].

Contraction and relaxation of the myometrium results from the cyclic depolarization and repolarization of the membranes of the muscle cells. Myometrial cells are gathered together by gap junctions composed of connexin proteins. Connexinproteins furnish pathways for the correct conduction of action potentials. In pregnancy, the cell-to-cell channels are low, indicating poor coupling and decreased electrical conduction. Thus, the uterine contractions are low and the pregnancy is maintained. The spontaneously contracting uterus at term and preterm has increased cell junctions forming the electrical syncytium that is necessary for effective contractions. So, the uterus may contract without neuronal control [**40**,**41**].

Thus, where in this molecular puzzle is the rationale for cerclage in prevention of preterm birth? 

## Cervical cerclage

History, ultrasound or physical exam indicated cerclage have been proved to significantly reduce preterm birth in singleton pregnancies and increase it in twin gestations [**42**,**43**]. Generally, the procedure is performed at 12 to 24/28 weeks of gestation. The management of cerclage is not based on large randomized trials. Screening and treatment of sexually transmitted infection has not been proven to improve the cerclage outcome; even so, our practice always includes Gram stain, microbiologic culture from cervix and vagina, leukocyte count and reactive C protein. Current evidence is insufficient to recommend antibiotic prophylaxis and tocolytic drugs administration for either prophylactic or emergency cerclage. We always treat by tocolysisany vaginal documented infection, including group B streptococcus and also superimposed uterine contractions; the Indomethacin administration always follows the procedure for 48 hours. Available data regarding progesterone supplementation is limited; we continue progesterone supplementation in women with a positive history for preterm birth and initiate the prophylaxis before the placement of the cerclage in women without antecedents of pregnancy loss or premature delivery. The object of cerclage placement is to reinforce the cervix at the level of the internal os; lengthening the cervix being the secondary aim. Berghelleat al. demonstrated that achieving a cerclage height >2 cm reduces preterm birth compared with shorter cervical height [**8**], but even a 10 mm cervical length was associated with delivery at a more advanced gestational age. The decrease of the physical activity is advisable after the procedure.

## Discussions and conclusions

What do we know by now? We know that preterm birth is considered one of the most important health problems in Obstetrics, having a continuous increased incidence despite significant medical advances acquired in the etiopathology, prediction, and therapeutic approach. We also know that the ultrasound evaluation of the cervix is predictive for preterm birth, but we do not have enough evidence to recommend it to be used routinely and generally in the prediction of an event,which is dependent on multiple variables that could act at any moment on a dynamic system. Moreover, we know that progesterone decreases preterm delivery and neonatal morbidity, and even if cerclage is effective in singleton gestations with prior preterm birth, prior second-trimester loss or when indicated by the physical exam, it does not prevent preterm birth in all women with short cervical length on transvaginal ultrasonography.

**Fig. 1:**
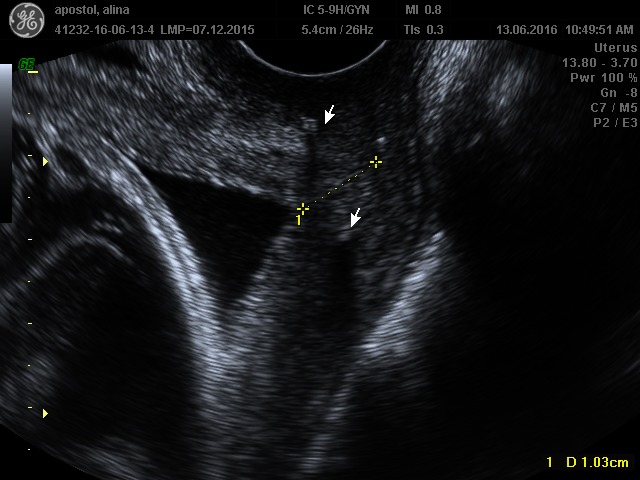
Patient A

**Fig. 2:**
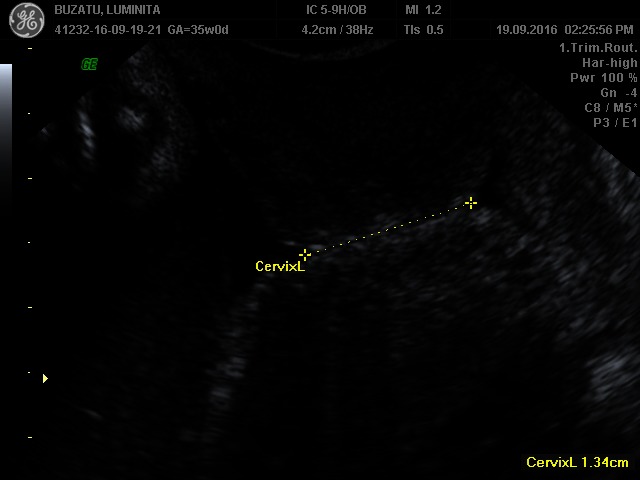
Patient B

From the cervical point of view, if we could distinguish between short cervix by insufficiency and short cervix by uterine contractions, we would be able to better predict and prevent preterm delivery. Thus, at present, we do not know which is the difference between patient A and patient B, and why the cervix with cerclage performed on prolapsed fetal membranes into vagina at 20 weeks of gestation, in the context of the absence of a suggestive history and of risk factors, had experienced a preterm birth at 37 weeks of gestational age, and why the short cervix with a preterm delivery in antecedents reached the term in the absence of cervical cerclage (**Fig. 1**,**2**).

In our experience, a transvaginal ultrasound screening for the measurement of the cervix is required. The proper gestational age for the prediction of preterm birth, based on length and appearance of the cervix,is considered to be between 18-22 weeks of gestation, and 12-16 weeks for singleton pregnancies with relevant history. Just from an observational point of view, for the moment, we can affirm that the cerclage of the cervix is unnecessary if the cervical length is above 2 cm and the internal cervical os is closed, taking into account that inserting the cerclage wire is usually done under this cut-off. In such cases, we observed a significant efficiency of the treatment with progesterone. We practice sustained progesterone and Indometacin cerclage only in the presence of funneling, convinced that the mechanism responsible for the cervix shortening with a closed internal os is the uterine contraction and not cervical insufficiency.

The question that remains open, among so many others, is the following: what are the anatomical or morphological events that cause cervical insufficiency in women with previously normal pregnancies term delivered?
